# About the Implementation of Frequency Conversion Processes in Solar Cell Device Simulations

**DOI:** 10.3390/mi9090435

**Published:** 2018-08-30

**Authors:** Alexander Quandt, Tahir Aslan, Itumeleng Mokgosi, Robert Warmbier, Maurizio Ferrari, Giancarlo Righini

**Affiliations:** 1 Historical Museum of Physics and Study & Research Centre “Enrico Fermi”, 00184 Roma, Italy; maurizio.ferrari@unitn.it (M.F.); giancarlo.righini@centrofermi.it (G.R.); 2 School of Physics and Materials for Energy Research Group, University of the Witwatersrand, 2050 Johannesburg, South Africa; 772891@students.wits.ac.za (T.A.); itu.mokgosi@gmail.com (I.M.); 3 Department of Physics, University of Johannesburg, 2006 Auckland Park, South Africa; rwarmbier@uj.ac.za; 4 Institute of Photonics and Nanotechnologies, National Research Council of Italy, 38123 Povo, Italy; 5 Nello Carrara Institute of Applied Physics, National Research Council of Italy, 50019 Sesto Fiorentino, Italy

**Keywords:** photovoltaics, frequency conversion, device simulations

## Abstract

Solar cells are electrical devices that can directly convert sunlight into electricity. While solar cells are a mature technology, their efficiencies are still far below the theoretical limit. The major losses in a typical semiconductor solar cell are due to the thermalization of electrons in the UV and visible range of the solar spectrum, the inability of a solar cell to absorb photons with energies below the electronic band gap, and losses due to the recombination of electrons and holes, which mainly occur at the contacts. These prevent the realization of the theoretical efficiency limit of 85% for a generic photovoltaic device. A promising strategy to harness light with minimum thermal losses outside the typical frequency range of a single junction solar cell could be frequency conversion using rare earth ions, as suggested by Trupke. In this work, we discuss the modelling of generic frequency conversion processes in the context of solar cell device simulations, which can be used to supplement experimental studies. In the spirit of a proof-of-concept study, we limit the discussion to up-conversion and restrict ourselves to a simple rare earth model system, together with a basic diode model for a crystalline silicon solar cell. The results of this show that these simulations are very useful for the development of new types of highly efficient solar cells.

## 1. Introduction

The conversion efficiencies of silicon-based solar cells are getting closer and closer to the theoretical limit of 34% for single-junction silicon-based solar cells, as estimated by Shockley and Queisser in 1961 [1]. These impressive numbers must, however, be compared to the theoretical efficiency limit of 85% for a generic photovoltaic device [2], which shows that there is plenty of room for improvements. The main losses in a typical semiconductor solar cell are due to the thermalization of electrons in the UV and visible range of the solar spectrum, the inability of such devices to absorb photons with energies below the electronic band gap, and losses due to the recombination of electrons and holes, which occur particularly at the contacts. A recent survey of novel design strategies to overcome these problems has been given by Polman and Atwater [3], but their main focus was on multi-junction solar cells. Another promising strategy for single junction solar cells to harness light outside their typical frequency ranges is frequency conversion using rare earth ions, as suggested by Trupke [4]. From a technical point of view, the most common way to introduce frequency conversion into a standard solar cell is through additional functional glass layers containing rare-earth ions. In device simulations of solar cells, we may cater for the new light management features through calculating the additional photon fluxes that result from the frequency conversion processes in these additional functional layers. 

In this work, we want to demonstrate this approach for a model solar cell. In this proof-of-concept approach, a standard solar cell model is augmented by adding the effects of an additional up-conversion (UC) glass layer. After describing some of the technical background related to up-conversion, we also highlight some of the relevant processes involved in typical UC applications for solar cells by using a rate equation model. We also explain in some detail how a typical device simulation is carried out, and we emphasize the crucial role of the size of the model glass layer for the performance of a model c-Si solar cell device.

## 2. Up-Conversion in Solar Cells

**Figure 1:**
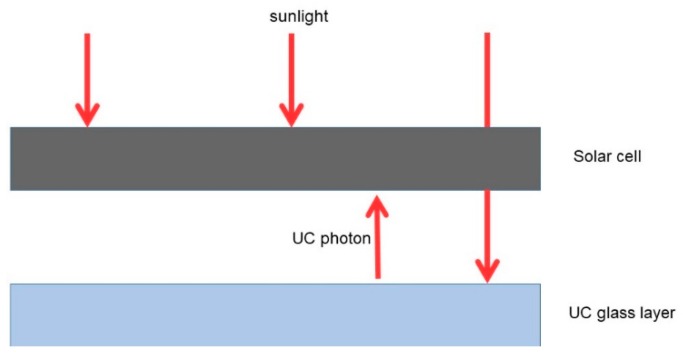
Schematic for up-conversion using glass layers in solar cell applications.

In the context of solar cells, up-conversion is used to absorb sub-band-gap photons from the solar spectrum and convert them into higher-energy photons in the normal absorption range of the solar cell. The up-converted photons can be utilized by the solar cell to produce electron-hole pairs, which drive the corresponding photocurrents [2]. Note that about 20% of the solar energy reaching the surface of the Earth is not utilized by conventional silicon solar cells, as these photons have energies smaller than the band gap of the semiconductors in the solar cell [5]. Therefore, UC provides an attractive method to harness these lower-energy photons, reducing spectral losses. This increases the photocurrent and improves the overall efficiency of the solar cell. A simplified diagram illustrating the UC process is given in Figure 1.

In general, the UC process requires a luminescent material with multiple energy levels that have an appropriate energy spacing. This allows for the emission of photons with frequencies close to the band gap of the solar cell. It is well-known that lanthanide ions, such as Er^3+^, have these properties, which can easily be embedded into glass layers for the purpose of UC [6,7]. One of the approaches that are currently being studied in Reference [8] is the systematic doping of optical materials with one or more of these so-called activator ions, with the possible inclusion of sensitizer ions, such as Yb^3+^, as co-dopants.

## 3. Current Densities and Generation Rate of Cell with Frequency Conversion

In the following, we briefly describe the main contributions to the current density generated by a solar cell. These can be divided into an electronic contribution from the P-N junction, the standard photocurrent, and an additional current from the up-converted photons.

### 3.1. Dark Current Density

If an external voltage is applied to a P-N junction, a net current density $J_{dark}$ will be obtained. This current density is generated under dark conditions and yields an important reference value. This current is given by Reference [9]:

(1) $$J_{dark\;}=\left(J_{diff,0\;}+\;J_{scr,\;0\;}+\;J_{rad,\;0}\right)\left(e^{\frac{qV}{kT}}-1\right)$$

where $J_{diff,0}$ is the diffusion current density, $J_{scr,0}$ is the recombination current density, and $J_{rad,0}$ is the irradiative recombination current density. k is the Boltzmann’s constant, T is the temperature of the device in Kelvin, and q is the electronic charge. The dark current is completely described by electronic semiconductor physics. 

### 3.2. Photo-Current Density

The photo current density $J_{p}$ stems from the free charge carriers generated through photon absorption and is given by [9,10]:

(2) $$J_{p}=q\eta_{c}\int_{\lambda_{1}\;}^{\lambda_{2}}(1-R(\lambda ))\alpha \left(\lambda \right)\exp\left[-\alpha \left(\lambda \right)X\right]\Phi_{\lambda }\left(\lambda \right)d\lambda$$

where $\eta_{c}$ is the charge carrier generation quantum efficiency which is equal to 1, α(λ) is the absorption coefficient, R(λ) is the reflectivity, X is thickness of the absorbing layer, and $\Phi_{\lambda }\left(\lambda \right)$ is the incident spectral photon flux at wavelength λ. In this work, we shall restrict ourselves to the standard AM1.5 spectrum as found in Reference [9]. Reflections and other losses are not included in this model. 

### 3.3. Additional Current Density from UC

In the case of solar cells with UC, an extra current density must be added to the standard photocurrent density in Equation (2). To this end, we estimate the additional photon flux ($\Phi_{ex}\left(d\right))$ due to frequency conversion, using:

(3) $$\Phi_{ex,\;i}\left(d\right)=\frac{N_{i}}{\tau_{i}}d$$

where $N_{i}$ denotes the population density of state i from which electrons decay to release upconverted photons. $\tau_{i}$ is the lifetime of this state, and d is the thickness of the frequency conversion layer. Equation (2) needs to be used with caution, as it does not consider extra losses which might be introduced by a thick conversion layer, e.g., extra reflection. It also assumes $N_{i}$ to be position independent, which also only holds for thin conversion layers.

The generation rate $G_{ex,i}$ accounts for the creation of electron-hole pairs in the solar cell from the extra photon flux $\Phi_{ex,\;i}$. It can be estimated from:

(4) $$\;G_{ex,i\;}=\alpha \left(\lambda_{0}\right)\;\Phi_{ex,i}\left(\lambda_{0}\right)$$

where $\alpha \left(\lambda_{0}\right)$ is the absorption coefficient of the solar cell at the up-converted wavelength. The extra current density produced by a single emission line from the up-conversion is then given by:

(5) $$J_{ex,i\;}=q\;G_{ex,i}L_{D}$$

where $L_{D}$ is the size of the depletion layer in the solar cell. 

The total current can then be estimated from the summation of the above current densities, and is given by:

(6) $$J_{tot\;}=\;J_{dark}+\;J_{p}+\;J_{ex,i}$$

if more than one UC channel exists, the last term in Equation (5) becomes a sum over the corresponding current density contributions.

The power conversion efficiency of a solar cell is defined by:

(7) $$\eta =\;\;\frac{V_{m}\times J_{m}\;}{P_{in}}$$

here, $P_{in}$ is the input solar power intensity, and $V_{m}$ and $J_{m}$ are the voltage and the current densities at the maximum power point of the solar cell. $V_{m}$ and $J_{m}$ can be easily determined from the current-voltage curve.

It should be noted that the above model is, of course, highly idealised, and many interactions between the UC layer and the rest of the solar cell are not treated. However, it is a strength of this model to treat the reference cell and the UC layer as separate systems, as this allows us to compute a qualitative estimate of the potential benefit of a UC layer to the solar cell efficiency without the necessity for a more cumbersome simulation.

In the following, we want to focus our discussion of UC on a simple-term scheme for trivalent Erbium (Er^3+^) and a rate equations-based approach.

## 4. Er^3+^ Up-Conversion Term Scheme

To describe up-conversion properly, four physical processes need to be considered, which are: ground state absorption (GSA), excited state absorption (ESA), spontaneous emission (SPE), stimulated emission (STE), and energy transfer (ET) [11]. Many lanthanides are suitable for up-conversion applications. Of those, the trivalent Erbium (Er^3+^) features a rather simple term scheme, which makes Er^3+^ a good study case without adding unnecessary complexity.

**Figure 2:**
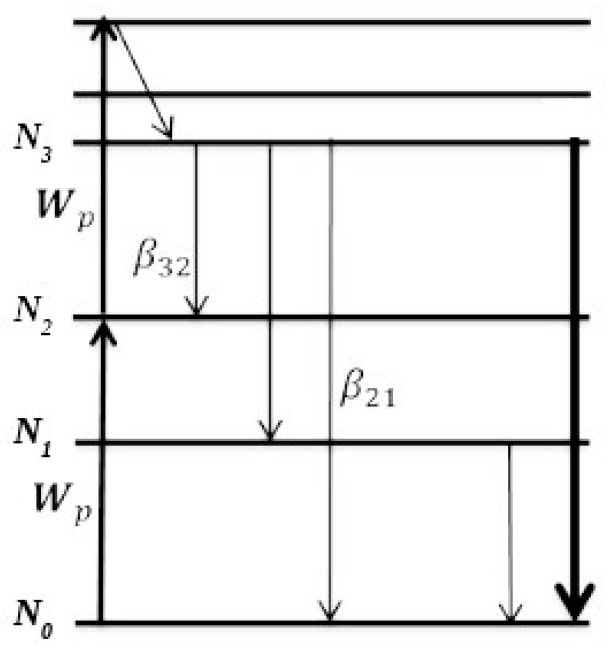
Energy level diagram for Er^3+^ with all the important processes for up-conversion, as described in Reference [12].

A Er^3+^ term scheme is shown in Figure 2, which only considers the processes and states relevant for UC. The processes of GSA and ESA are assumed to be resonant. Three metastable states are shown, from which some further electronic transitions (accompanied by the emission of photons) originate with branching ratios *β_ij_*. Here, *i* denotes the initial state of such a transition, whereas *j* denotes the final state [12].

The rate equations for this basic system are given by [12]:

(8) $$\frac{dN_{3}\;}{dt}=\frac{\sigma_{ESA}}{\sigma_{ESA}+\sigma_{GSA}}W_{p}N_{2}-\;\frac{N_{3}}{\tau_{3}}$$

(9) $$\frac{dN_{2}\;}{dt}=\frac{\sigma_{GSA}}{\sigma_{ESA}+\sigma_{GSA}}W_{p}N_{0}-\;\frac{N_{2}}{\tau_{2}}-\frac{\sigma_{ESA}}{\sigma_{ESA}+\sigma_{GSA}}W_{p}N_{2}+\;\beta_{32}\frac{N_{3}}{\tau_{3}}$$

(10) $$\frac{dN_{1}\;}{dt}\;=\;\beta_{31}\frac{N_{3}}{\tau_{3}}\;+\;\beta_{21}\frac{N_{2}}{\tau_{2}}\;-\;\frac{N_{1}}{\tau_{1}}$$

(11) $$N_{tot\;}=\;N_{0}+\;N_{1}+\;N_{2}+\;N_{3}$$

$W_{p}$ is the pump rate used to populate the excited states. In the case of solar cells, $W_{p}=\sigma_{P}\Phi_{p}$ is given by the incoming photon flux $\Phi_{p}\approx 1.2\times 10^{17}\;{\mathrm{cm}}^{-2}s^{-1}$ around the up-conversion wavelength, as well as the absorption cross-section $\sigma_{p}\approx 1.0\times 10^{19}{\;\mathrm{cm}}^{2}$ [13]. The exact line position and line width depend on the host material of the UC layer, meaning this approximation shall suffice here. $N_{tot}$ is the total population density of the active erbium ions within the up-conversion glass layer. $\sigma_{i}$ and $\tau_{i}$ are the cross sections and lifetimes for state i, respectively. $\beta_{ij}$ denotes the branching ratio from state i to state j. $N_{0}$, $N_{1}$, $N_{2}$, and $N_{3}$ are the population densities for various states of the Er^3+^ ion.

Equations (8)–(10) describe the population changes of the meta-stabile excited states, while Equation (11) guarantees conservation of electrons. An extra equation for the ground state population $N_{0}$ is not needed, as the system of equations is later solved in the steady-state case, for which the system is already complete. Equation (8) includes the change in $N_{3}$ through pumping from $N_{2}$, as well the emission. The population of $N_{2}$ is fed by pumping from the ground state $N_{0}$ and emission from $N_{3}$, and loses population through pumping to $N_{3}$ and emission. $N_{1}$ is populated through emissions from $N_{2}$ and $N_{3}$, and emits itself back to the ground state.

## 5. Results and Discussions

**Figure 3:**
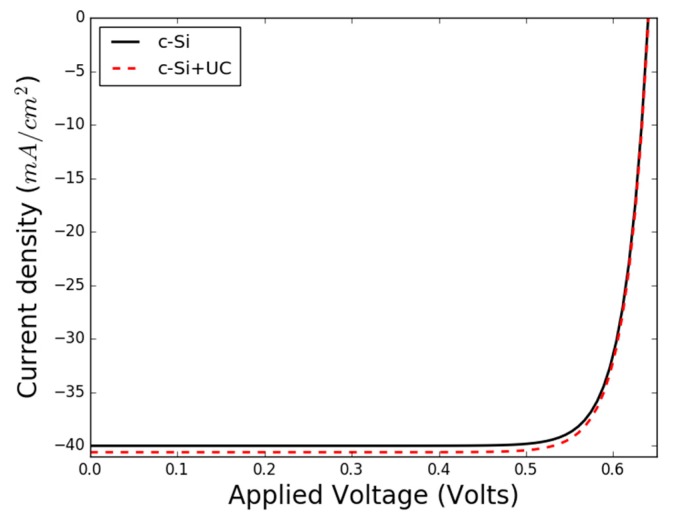
Simulated J–V curve for an ideal c-Si solar cell with and without up-conversion (UC).

A reference 1-D crystalline silicon (c-Si) solar cell is modelled using Equations (1) and (2) [9,10]. For the calculation of $J_{dark}$ in Equation (1), the sum of the different current densities was specified as $J_{diff,0}+\;J_{scr,0}+\;J_{rad,0}=1.95\times 10^{-9}\;A/m^{2}$. The operating temperature of the device was chosen to be 300 K. $J_{p}$ is estimated by computing Equation (2), where the absorber thickness X was chosen to be $\;510\times 10^{-6}\;m$. The resulting I–V curve for this model solar cell is given by the solid curve in Figure 3. Alternatively, a reference solar cell which can be modelled by various device simulation packages such as GPVDM [12] can be used. In this approach, the resulting photon fluxes from the UC layer are used to augment the photon flux values on the actual spectrum file in the program codes. We stick to using our reference solar cell model for this work. 

In the steady state, the population densities $N_{0}$, $N_{1}$, $N_{2},$ and $N_{3}$ do not change over time, and can be calculated as a function of the pump power $W_{p}$, provided $N_{tot}$ and all necessary constants in Equations (8)–(11) are known. The important parameters needed to solve for the population densities are given in Table 1.

In the Er^3+^ term scheme, $N_{3}$ leads to photoemission with 550 nm wavelength, which is the relevant transition for up-conversion applications. The corresponding lifetime $\tau_{3}$ for $N_{3}$ is 0.37 ms [13]. The values in Table 1 are used to estimate the additional current density from the UC material layer. All the parameters for crystalline silicon used in the subsequent calculations are taken from [9].

The generation rate $G_{ex,3}$ from the up-converted photons is calculated using an absorption coefficient of $\alpha \approx 10^{6}\frac{1}{m}$ for c-Si at 550 nm. With this, the photon flux calculated from Equation (3) is $\Phi_{ex,\;3}\approx 5.35\times 10^{19}\;\frac{1}{s\;\times {\;m}^{2}}$. Using Equation (8), the corresponding generation rate is obtained as $G_{ex,3}\approx 5.35\times 10^{25}\;\frac{1}{s\;\times {\;m}^{3}}$. This should be compared to the generation rate $G_{Si}\approx 4.48\times 10^{27}\;\frac{1}{s\;\times {\;m}^{3}}$ of a c-Si solar cell without UC. 

It is important to note that the estimated photon flux strongly depends on the size of the glass layer. A very thick glass layer, like the present one, may strongly exaggerate the additional photon flux due to UC. Also, for very thick glass layer attenuation (Beer’s law), the additional photon flux needs to be considered. Therefore, our guideline is to use glass layers of a size that leads to measurable increases in overall efficiency (i.e., in the range of %), but with the final aim of identifying optimum UC systems, where these glass layers may be kept very small.

A certain increase in photon flux does not imply a proportional increase in solar cell efficiency, because the solar cell is a nonlinear device. It is necessary to calculate the corresponding current densities. Using Equation (5), we may determine the additional photocurrent density derived from the UC process. The total current density can then be determined by using Equation (6). The resulting J–V curves are shown in Figure 3, for a c-Si solar cell with (solid black curve) and without (dashed red curve) the UC current density contribution.

Without the frequency conversion layer, the efficiency of the model c-Si solar cell is 21.4%, with a short circuit current of $J_{sc}=400\;\frac{A}{m^{2}}$ and an open circuit voltage of $V_{oc}=0.56\;V.$ Using Equation (5), the additional current density that is derived from the UC glass layer is $J_{ex,3}=8\;\frac{A}{m^{2}}$. This results in a total short-circuit current of $J_{sc+ex}=408\;\frac{A}{m^{2}}$. The open circuit voltage is not affected. 

The efficiency of the model c-Si solar cell, including contributions from UC, is 22.7%. The overall increase in efficiency for the c-Si solar cell by adding our Er^3+^-based luminescent glass layer model is 1.3%. The increase in efficiency in this proof-of-concept example is, of course, small. It stems from one specific up-conversion channel only, and the example did not include any sensitizer co-dopants. More importantly though, this modelling approach can be applied to more complex and realistic up-conversion set-ups to find good up-conversion configurations.

Some experimental work has been done studying the impact of frequency conversion, such as UC, on solar cell device performance. In their recent publication, Kumar et al. [14] found a UC improvement of about 0.4% for a dye-sensitized solar cell that uses Er^3+^ and Yb^3+^ co-doped ZnO up-conversion (UC) nanoparticles-based phosphors. Grigoroscuta et al. [15] have also studied the effect of a phosphor film of Yb^3+^/Er^3+^-co-doped CeO_2_ on the performance of a silicon-based solar cell and have noticed a significant increase in the cells’ performance because of the thin UC film. A simplified modelling approach such as this can be useful in aiding the development of more advanced solar cells.

## 6. Conclusions

In this paper we presented a simple model approach to estimate the impact of frequency conversion on the overall efficiency of solar cell devices. A rate equation model was used to estimate the population densities for a typical UC process in Er^3+^ yielding an extra photon flux in the visible range. The total photocurrent is enhanced by the addition of an up-conversion layer. As the solar cell is a non-linear device, already relatively small increases in the current density can lead to considerable improvements in the overall device efficiency.

Systems other than Er^3+^ can be studied using the same method with Tm^3+^ being a good example, provided the basic parameters for the rate equation model are known. As an alternative to the present approach, the additional photon flux due to UC can also be used as a direct input parameter for existing device simulation codes [16], which seems to compare quite well to the presented approach. 

## Figures and Tables

**Table 1:** Parameters for solving the rate equations. (* obtained from Reference [13]).

Parameter	Symbol	Value
*Lifetime	$\;\tau_{1}$	10 ms
*Lifetime	$\;\tau_{2}$	4.3 ms
*Lifetime	$\;\tau_{3}$	0.37 ms
*GSA cross section	$\;\sigma_{GSA}$	0.25 × 10^−20^ cm^2^
*ESA cross section	$\;\sigma_{ESA}$	1.7 × 10^−20^ cm^2^
*Branching ratio	$\;\beta_{20}$	0.85
*Branching ratio	$\;\beta_{21}$	0.15
*Branching ratio	$\;\beta_{30}$	0.67
*Branching ratio	$\;\beta_{31}$	0.27
*Branching ratio	$\;\beta_{32}$	0.02
Pump wavelength	$\;\lambda_{p}$	974 nm
Conversion material length	d	0.3 mm
*Total Er^3+^ concentration	$\;N_{tot}$	1 × 10^19^ cm^−3^
Depletion layer width	$\;L_{D}$	1 × 10^−6^ m
